# Proteome turnover in the bloodstream and procyclic forms of *Trypanosoma brucei* measured by quantitative proteomics

**DOI:** 10.12688/wellcomeopenres.15421.1

**Published:** 2019-10-09

**Authors:** Michele Tinti, Maria Lucia S. Güther, Thomas W. M. Crozier, Angus I. Lamond, Michael A. J. Ferguson

**Affiliations:** 1 The Wellcome Centre for Anti-Infectives Research, School of Life Sciences, University of Dundee, Dundee, UK; 2 Centre for Gene Regulation and Expression, School of Life Sciences, University of Dundee, Dundee, UK; 3 Department of Medicine, Cambridge Institute for Medical Research, Cambridge, UK

**Keywords:** Turnover, Proteomics, Trypanosoma, Bloodstream, Procyclic

## Abstract

**Background**: Cellular proteins vary significantly in both abundance and turnover rates. These parameters depend upon their rates of synthesis and degradation and it is useful to have access to data on protein turnover rates when, for example, designing genetic knock-down experiments or assessing the potential usefulness of covalent enzyme inhibitors. Little is known about the nature and regulation of protein turnover in *Trypanosoma brucei*, the etiological agent of human and animal African trypanosomiasis.

**Methods**: To establish baseline data on *T.* *brucei* proteome turnover, a Stable Isotope Labelling with Amino acids in Cell culture (SILAC)-based mass spectrometry analysis was performed to reveal the synthesis and degradation profiles for thousands of proteins in the bloodstream and procyclic forms of this parasite.

**Results**: This analysis revealed a slower average turnover rate of the procyclic form proteome relative to the bloodstream proteome. As expected, many of the proteins with the fastest turnover rates have functions in the cell cycle and in the regulation of cytokinesis in both bloodstream and procyclic forms. Moreover, the cellular localization of *T. brucei* proteins correlates with their turnover, with mitochondrial and glycosomal proteins exhibiting slower than average turnover rates.

**Conclusions**: The intention of this study is to provide the trypanosome research community with a resource for protein turnover data for any protein or group of proteins. To this end, bioinformatic analyses of these data are made available via an open-access web resource with data visualization functions.

## Introduction

The tsetse transmitted protozoan parasites of the species complex *Trypanosome brucei* cause human African trypanosomiasis (HAT) and the cattle disease Nagana ^1^. The parasites undergo a complex lifecycle between their mammalian hosts and insect vectors. The parasites multiply as procyclic forms in the tsetse midgut, some of which migrate to the salivary glands where they differentiate to epimastigote forms and then into non-dividing metacylic trypomastigote forms. The latter are transmitted to the host during the insect bloodmeal, where they differentiate in into rapidly dividing slender trypomastigotes that colonise primarily the hemolymphatic system. Some parasites differentiate into non-dividing stumpy trypomastigotes that are pre-adapted to differentiate into procyclic forms if taken up by a tsetse in a bloodmeal, thus completing the lifecycle. It is the passage of slender trypomastigote forms across the blood-brain barrier that leads to the severe neutrological symptoms of HAT. During this complex lifecycle, *T. brucei* achieves differentiation and responds to changes in its environment through adaptation of its proteome ^2– 9^. With the aim of gaining further insights into the proteome plasticity of this parasite, we set out a mass spectrometry strategy to define the global proteome turnover rate of *T. brucei* and compare the results between the BSF and PCF lifecycle stages.

## Methods

### Cell culture

PCF *Trypanosoma brucei* clone 29.13.6 cells, kindly provided by Prof. George Cross, were maintained in original SDM-79 (Invitrogen, Trypanosome Community, UK) containing 10% heat-inactivated fetal bovine serum (Gibco, Invitrogen), 2 g/L sodium bicarbonate, 2 mM Glutamax I (Invitrogen, UK), 7.5 mg/L haemin, 15 μg/ml G418 and 50 μg/ml hygromycin at 28°C without CO _2_ (non-treated plastic culture flask, Thermo Scientific). Culture adapted strain 427 monomorphic BSF *T. brucei* (variant 221, MITat 1.2) genetically modified to express T7 RNA polymerase and tetracycline repressor protein, known as Single Marker cells ^10^, were cultured in HMI-9T medium, a modification of the original HMI-9 which contains 56 μM 1-thioglycerol instead of 200 μM 2-mercaptoethanol, 10% heat-inactivated fetal bovine serum (Gibco, Invitrogen), 2 mM Glutamax I, 2.5 μg/mL G418 at 37°C with 5% CO _2_.

### Stable isotope labelling with amino acids in cell culture (SILAC) label-chase cell cultures

All isotopes were from CK Isotopes, Cambridge Isotope Labs, USA. *T. brucei* PCF cells were labelled to steady state, i.e., during 8 to 9 cell divisions, using medium isotope SILAC SDM-79 medium, containing [U- ^13^C]-L-arginine.HCl and [ ^2^H _4_]-L-lysine.2HCl, known as R6K4, as described before ^4, 11^. Cells were recovered by centrifugation (600 × g for 10 min at room temperature) and transferred into pre-warmed (27°C) light isotope SDM-79 medium (chase medium), containing unlabelled L-arginine.HCl and L-lysine.2HCl, known as R0K0, to a final cell density of 1 × 10 ^7^ cells/ml. Aliquots of 3 × 10 ^7^ cells were removed in triplicate 0.25, 0.5, 1, 2, 4, 8, 20, and 28 h after transfer to chase medium. The 0-h chase time point was taken in triplicate just before cells were transferred to the light (R0K0) chase medium. Cell aliquots from each time point were placed on ice-water, counted using a Z2 Coulter counter (Beckman) and immediately diluted with 10 ml ice-cold PBS to stop the chase. The cells were then centrifuged (800 × g for 10 min at 4°C) and resuspended in 0.1 ml PBS with subsequent addition of equal volume of lysis buffer (freshly prepared 4% SDS in 0.1 M Tris-HCl pH 7.2 containing 0.1 M DTT), vortexed and heated for 20 min at 50°C. Aliquots were snap-frozen in liquid nitrogen and stored at -80°C until further processing. Before filter-aided sample preparation (FASP; see below), cell lysates from each time point were mixed 1:1 with equivalent lysates from cells labelled to steady-state using heavy isotope SILAC SDM-79 medium, containing [ ^13^C _6_, ^15^N _4_]-L-Arginine.HCl and [ ^13^C _6_, ^15^N _2_]-L-Lysine.2HCl, known as R10K8.

*T. brucei* BSF cells were labelled to steady state levels, between 8 to 9 cell divisions, using medium isotope (R6K4) SILAC HMI-11T medium, containing 120 μM [U-13C]-L-Arginine.HCl and 240 μM [ ^2^H _4_]-L-Lysine.2HCl as previously described ^4^. When the culture reached 1 × 10 ^6^ cells/ml, the labelled cells were harvested by centrifugation at room temperature and transferred into light isotope (R0K0) HMI-11T chase medium, containing 399 μM L-Arginine.HCl and 800 μM L-Lysine.2HCl, at a final cell density of 6.2 × 10 ^5^ cells/ml. Aliquots of 1.5 × 10 ^7^ cells were removed in triplicate at 0.5, 1, 2, 4, 8 and 12 h after transfer to chase medium. The 0 h chase time point was taken in triplicate just before cells were transferred to the light (R0K0) chase medium. Cell aliquots from each time point were placed on ice-water, counted in using Z2 Coulter counter (Beckman) and immediately diluted with 10 ml ice cold PBS to stop the chase. The cells were then centrifuged (800 × g for 10 min at 4°C) and resuspended in 0.05 ml PBS with subsequent addition of equal volume of lysis buffer (freshly prepared 4% SDS in 0.1 M Tris-HCl pH 7.2 containing 0.1 M DTT), vortexed and heated for 20 min at 50°C. Aliquots were snap frozen in liquid nitrogen and stored -80°C until further processing. Before FASP, see below, cell lysates from each time point were mixed 1:1 with equivalent lysates from cells labelled to steady-state in heavy isotope (R10K8) SILAC HMI-11 medium.

### FASP

Tryptic peptides were prepared using the FASP method ^12^ with minor modifications. Briefly, BSF and PCF samples were defrosted, vortexed, reduced with 100 mM DTT by heating at 50°C for 20 min and combined 1:1 to the corresponding R10K8 labelled BSF or PCF lysate. Samples were mixed with 8 M urea in 0.1 M Tris-HCl pH 8.5, placed inside spin filters (Vivacon 500, 10,000 MWCO) and washed with this buffer as described in the original protocol. Alkylation was performed using 50 mM iodoacetamide (freshly prepared) in the same buffer for 20 min at room temperature in the dark. Further washes with 8 M urea buffer, followed by washes with 50 mM ammonium bicarbonate buffer, were performed as in the original protocol. Digestion with trypsin (trypsin modified, sequencing grade, Roche) was performed with enzyme protein ratio 1:100 overnight at 37°C inside a humid chamber. Tryptic peptides were eluted by centrifugation into low binding Eppendorf tubes. Spin filters were washed with 50 μL 0.5 M NaCl, combined with the first eluate and acidified with 10 μL 10% trifluoroacetic acid (TFA), then further diluted with 400 μL of 0.1% TFA, desalted using C18-RP microspin silica columns (Nest group, USA) and freeze-dried.

### Fractionation of tryptic peptides and peptide LC-MS/MS analysis using MaxQuant

Aliquots of 50 µg of BSF and PCF freeze dried and desalted SILAC labelled tryptic peptides were re-dissolved in 50 µL of 5% formic acid and fractionated on an Xbridge BEH C18 column (130 Å, 3.5 µm, 4.6 × 150 mm) using a Dionex Ultimate 3000 HPLC system. Buffer A was composed of 2% acetonitrile in 10 mM ammonium formate (pH 9.0) and buffer B of 80% acetonitrile in 10 mM ammonium formate (pH 9.0). Columns were run at 1 mL/min at 30°C with a starting composition of 10% buffer B at 0 min, followed by an increase to 40% buffer B at 11 min, 100% at 12 min and down to 10% at 13 min until the end of the run at 20 min. Fractions (1 ml) were collected and subsequently pooled into 10 final fractions. The first 3 fractions were mixed with the final 3 fractions (i.e., 1 with 13, 2 with 12 and 3 with 11) dried using a GeneVac evaporator and redissolved in 50 µL of 5% formic acid. These peptide fractions in 5% formic acid were injected onto a C18 nano-trap column using a Thermo Scientific Ultimate 3000 nanoHPLC system, washed with 2% acetonitrile, 0.1% formic acid and resolved on a 150 mm × 75 μm C18 reverse phase analytical column using a gradient from 2% to 28% acetonitrile over 120 min at a flow rate of 200 nL/min. Peptides were ionised by nano-electrospray ionisation at 2.5 kV. Tandem mass spectrometry analysis was carried out on a QExactive+ mass spectrometer, using HCD fragmentation of precursor peptides. A data-dependent method was utilised, acquiring MS/MS spectra for the top 15 most abundant precursor ions.

### MaxQuant analysis

Data was processed using MaxQuant version 1.5.8.3, which incorporates the Andromeda search engine ^13^. Proteins were identified by searching a protein sequence database containing T. brucei brucei 927 annotated proteins (Version 32, downloaded from TriTrypDB ^14^) supplemented with frequently observed contaminants (porcine trypsin, bovine serum albumins and human keratins) and the Tb427.BES40.22 VSG protein as internal control. Search parameters specified an MS tolerance of 5 ppm, an MS/MS tolerance at 0.5 Da and full trypsin specificity, allowing for up to three missed cleavages. Carbamidomethylation of cysteine was set as a fixed modification and oxidation of methionine and N-terminal protein acetylation were allowed as variable modifications. Peptides were required to be at least 6 amino acids in length, and false discovery rates (FDRs) of 0.01 were calculated at the level of peptides, proteins and modification sites based on the number of hits against the reversed sequence database. A minimum of two peptides were quantified for each protein.

### Bioinformatic pipeline

The output proteinGroup file of the MaxQuant program was used to extract the data. The protein groups annotated by the MaxQuant program as ‘Only identified by site’, ‘Reverse’ and ‘Potential contaminant’ were removed from the analysis. Moreover, protein groups identified with less than 2 unique peptides were removed from the analysis. To calculate the incorporation rate, we analysed the peptide.txt output file of MaxQuant as described in 15 at section 12. Briefly, we first distinguished between lysine- and arginine-containing peptides then, for each of these subsets, we determined the incorporation rate as 1–1/average ratio of the Heavy and Medium labels versus the Light label, using the non-normalized ratios outputs of MaxQuant. The SILAC ratios R6K4/R10K8 (simplified as M/H) for degradation and R0K0/R10K8 (simplified L/H) for synthesis were extracted for each time point. The M/H and L/H values where normalized such that M/H+L/H=1 by computing M/H=M/H/(M/H+L/H) and L/H=L/H/(M/H+L/H). It is important to note that this transformation normalises for any sampling artefacts. The zero-hour time points of BSF and PCF were used to normalise the BSF degradation values to take into account incomplete steady-state incorporation of the heavy and medium isotopes with the formula:

 M/H’ = M/H × M/H _pcf _/ M/H _bsf_

Where M/H _bsf _and M/H _pcf _are the median M/H values at 0 hour for BSF and PCF proteome respectively.

To monitor parasite division, we determined the parasite density at each time point ( *Extended data*, Table 1 ^16^). Each degradation curve was fitted with an exponential decay model defined as described in Boisvert *et al*. 2012 and Ly *et al*. 2018 ^17, 18^:

*y* = amplitude × exp(- *x*/tau’)+offset

with the limfit Python library that uses non-linear least squares ^19, 20^. The amplitude, offset and tau’ parameters were initialised with the values of 0.9, 0.2 and 2, respectively. The parameters were set with a lower bound of zero. We used a weighted fitting, meaning that data points with smaller standard deviation had more importance for the curve fitting. The root mean square error (RMSE) between the estimated values and the predicted values were computed for all the fitted curves and reported to evaluate the quality of the fitting ( *Extended data*, Tables 2 and 3 ^16^). The half-life of the exponential decay models was computed by taking account of the cell division time. To this aim the tau ^’^ was corrected with the formula:

$$\text{tau}\;=\frac{1}{\frac{1}{\text{tau}\prime }\;-\;\frac{\text{ln}2}{\text{cdt}}}$$

were ln2 is the natural logarithm of 2 and cdt is the cell doubling time. The reported half-life was than computed as:

$$\text{half}\;\text{life}\;=\;-\text{tau}\;*\;\text{ln}\frac{\text{amplitude}\;-\;\text{offset}}{\text{amplitude}\;*\;2}$$

The BSF and PCF protein abundance was extracted by averaging the H Intensity (summed extracted ion current, XIC) values of the time course experiments for each protein. The Spearman correlation coefficient was used to compute correlation matrix between each data point of the BSF and PCF experiments. The Pearson correlation coefficient and the r ^2^ value was computed to compare the half-life values for the proteins in common between BSF and PCF cells. The Pearson and Spearman correlation coefficient and the r ^2^ values were computed with the scipy python package ^21^. The degradation linear motifs (degrons) were downloaded from the ELM database ^22^. The regular expressions (REs) corresponding to the degrons were searched for in the primary protein sequence with a custom Python code. The half-life values were binned into decile groups. The degron REs were searched for in the 1st and 2nd decile groups for proteins with short half-lives and in the 9th and 10th decile groups for proteins with long half-lives. For each degron, we reported the fraction of proteins with at least one RE match in each decile bin under analysis.

The Gene Ontology (GO) term enrichment analysis was computed with the GOATOOLS python package, version 0.8.12 ^23^ and the protein to GO term annotation was downloaded from TriTrypDB version 41 ^14^. Only GO terms with a p-value less than 0.01 and a minimum of 10 proteins annotated were reported. The localization information of the TrypTag project ^24^ was retrieved from the TriTrypDB web site using the download function of a pre-configured table and selecting Cellular Localization and Protein targeting and localization. The downloaded text file was parsed with a python script to extract the localization annotation from the HTML lines. For data visualization, only cellular compartments with more than 30 proteins annotated were used. Cell cycle regulated proteins were retrieved from Crozier *et al.* ^25^. Only proteins with a fold change value greater than 1.3 were selected (see Supplementary Table 1 of Crozier *et al.* ^25^). The predicted protein complexes were retrieved from Crozier *et al*. ^26^. We used the cumulative distribution of the half-life variance of proteins within the same predicted protein complexes and the residual protein amount variance of random complexes (the random complexes were equal in number and in size to the predicted protein complexes). The comparison was then repeated 1000 times, each time using a different seed for randomisation. We visualized the cumulative distribution of the variance between the predicted protein complexes and the random complexes and computed the p-value of the Kolmogorov-Smirnov test between the variances in the predicted and random protein complexes.

The Python code to reproduce the analysis pipeline and the figures reported in this paper are available as a series of Jupyter notebooks at https://github.com/mtinti/wor_turnover and on Zenodo ^27^.

## Results

**Figure 1.**
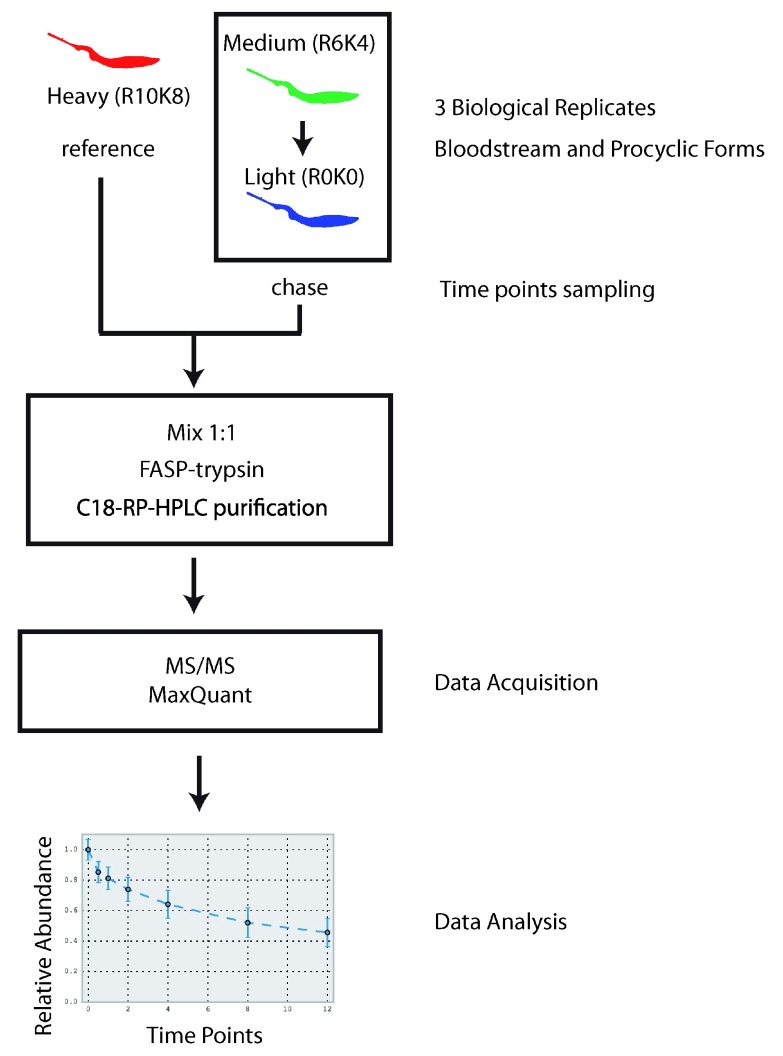
Experimental design. The flow chart summarises the experimental strategy used in this work to determine *T. brucei* bloodstream form (BSF) and procyclic form (PCF) proteome-wide turnover data.

**Figure 2.**
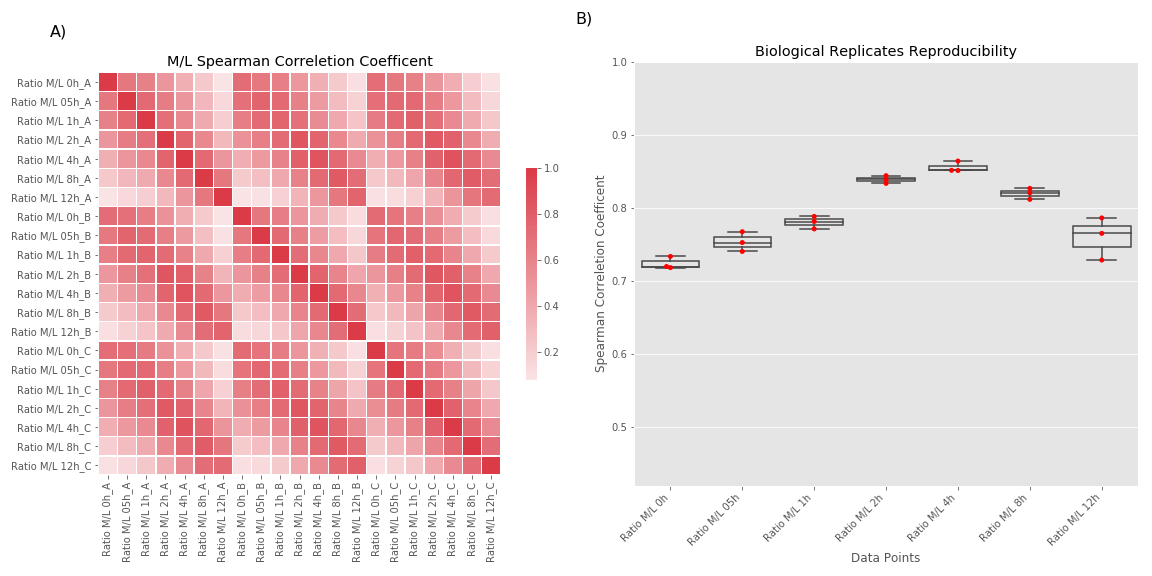
Correlation Analysis of BSF experiment. The figure analyses the experimenthal reproducibility of the BSF dataset. ( **A**) The panel reports an heatmap of the parwise Spearman correletion coefficents computed for all analysed time points (X- and Y-axis). ( **B**) The panel reports the parwise values of the Spearman correletion coefficents (Y-axis) for each time point (X-axis) of the 3 biological replicates.

**Figure 3.**
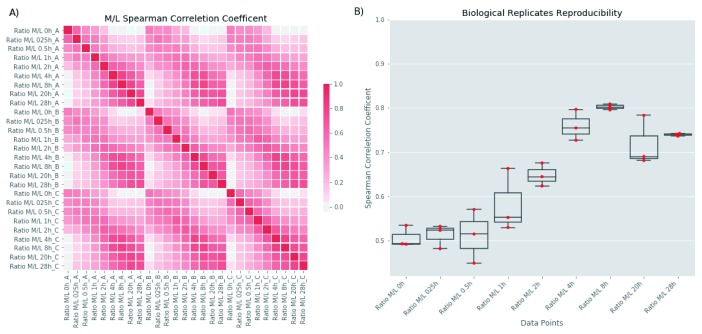
Correlation analysis of procyclic form (PCF) experiment. The figure analyses the experimenthal reproducibility of the PCF dataset. ( **A**) The panel reports an heatmap of the parwise Spearman correletion coefficents computed for all analysed time points (x and y-axis). ( **B**) The panel reports the parwise values of the Spearman correletion coefficents (y-axis) for each time point (x-axis) of the 3 biological replicates.

A SILAC label-chase technique was used to study the turnover rate of the *T. brucei* bloodstream (BSF) and procyclic forms (PCF) proteomes ( Figure 1). Triplicate biological replicates were analysed for both BSF and PCF cultures. For BSF parasites, cells were grown in parallel in heavy (H) and medium (M) L-Arg and L-Lys containing media. After 8 to 9 cell divisions, the steady-state medium-labelled parasites were placed in light (L) culture media and aliquots were taken at times of 0, 0.5, 1, 2, 4, 8, and 12 h and the cells lysed in an SDS-Tris buffer. Each time-point lysate was mixed 1:1 with corresponding heavy labelled lysate to provide an internal standard for normalization of synthesis and degradation data ^17^. Thus, the decrease of medium labelled peptides and increase of light labelled peptides relative to (constant) heavy labelled peptides over time were used to calculate rates of protein degradation and synthesis, respectively. Accurate cell counting was performed throughout the experiments to take into account changes in medium and light peptides relative to heavy peptides due to cell division during the chase period. Samples from each chase time-point were processed to tryptic peptides by FASP, separated into 10 sub-fractions by high-pH reversed-phase HPLC and analysed by LC-MS/MS. The same experiments were performed for PCF cells except that chase-times of 0, 0.25, 0.5, 1, 2, 4, 8, 20 and 28 h were used. In both cases, tryptic peptides were quantified using MaxQuant as described before ^4, 11, 13, 28^ and processed with an in-house developed pipeline. The MaxQuant program detected 6456 and 6466 protein groups in the BSF and PCF samples, respectively. These were filtered to remove protein groups annotated as: Only identified by site, Reverse hits and Potential contaminants. Further, we removed protein groups identified with less than two unique peptides. We evaluated the reproducibility of the biological replicates by computing the Spearman correlation coefficient between each data point of the BSF ( Figure 2A) and PCF ( Figure 3A) time course experiments using the M/L ratio of the MaxQuant protein group output. As expected, data points closer in time have a higher Spearman correlation coefficient relative to data points distant in time, creating the diagonal pattern visualized in the heatmaps of Figure 2A and Figure 3A. The Spearman correlation coefficient values between the time points of the 3 biological replicates is further visualized in Figure 2B for the BSF and Figure 3B for the PCF. Finally, we extracted normalized degradation profiles for 6023 and 5895 protein groups in the BSF and PCF respectively as described in the *Methods* section.

### Comparison of protein turnover between BSF and PCF parasites

**Figure 4.**
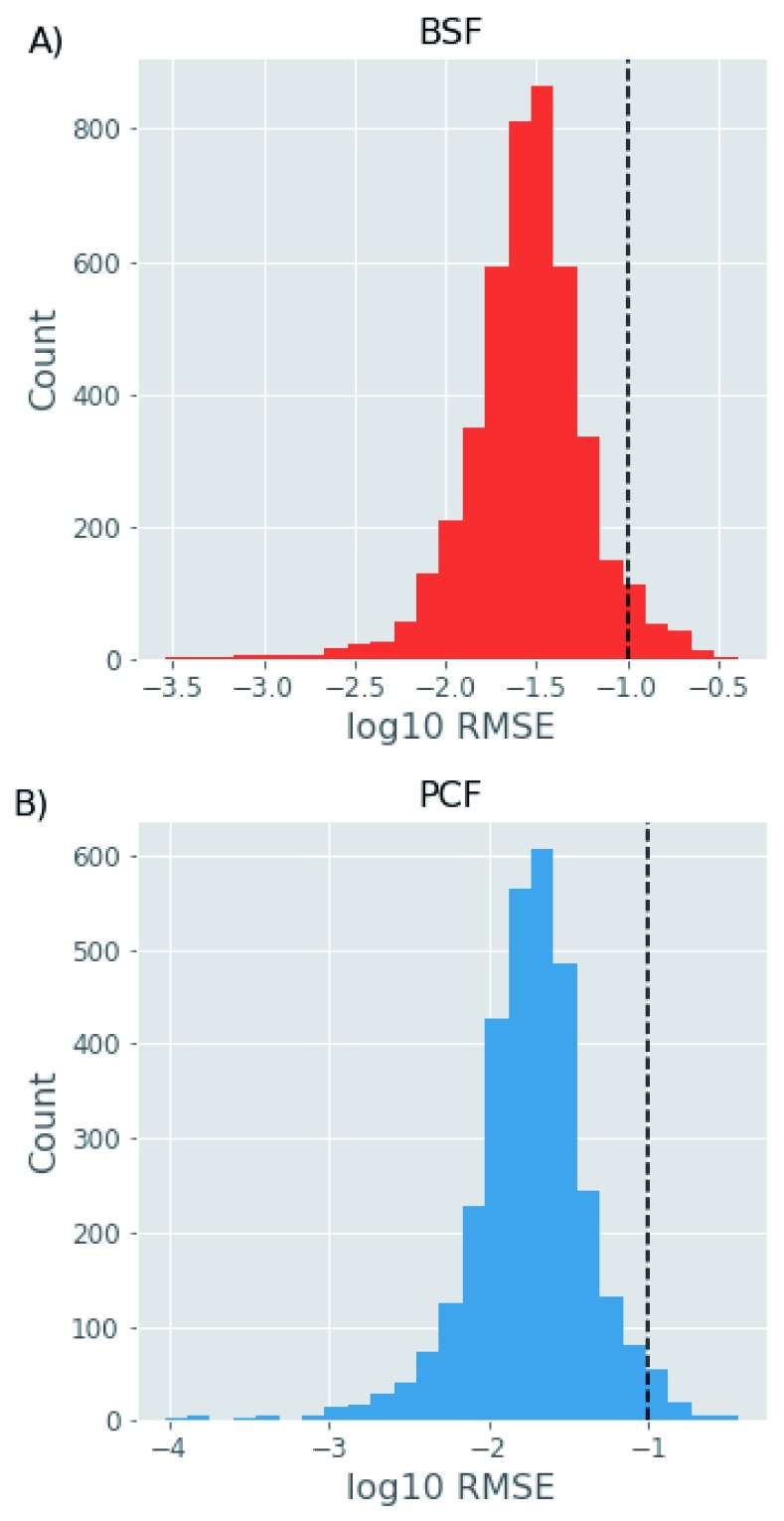
Quality threshold. The histograms show the log10 root-mean-square error (RMSE) values of the fitted exponential and linear models for the bloodstream form (BSF) ( **A**) and procyclic form (PCF) ( **B**) protein decay curves on the x-axis against the number of proteins within each log10 RMSE range. The dashed black line in each panel shows the threshold of 0.05 used to accept (to the left) or reject (to the right) the fitted models.

For the comparison of degradation profiles between BSF and PCF we filtered the data to include only protein profiles with good quality and reproducibility of the fitting outputs. Thus, we only analysed protein groups with at least two independent measurements in each of 4 time points for BSF and PCF cells. We also applied a threshold of 0.1 for the RMSE of the curve fit ( Figure 4). This final filtering step produced a dataset for 4194 protein groups in the BSF and 3092 protein groups in the PCF, with 2600 protein groups in common between the two life-cycle stages.

**Figure 5.**
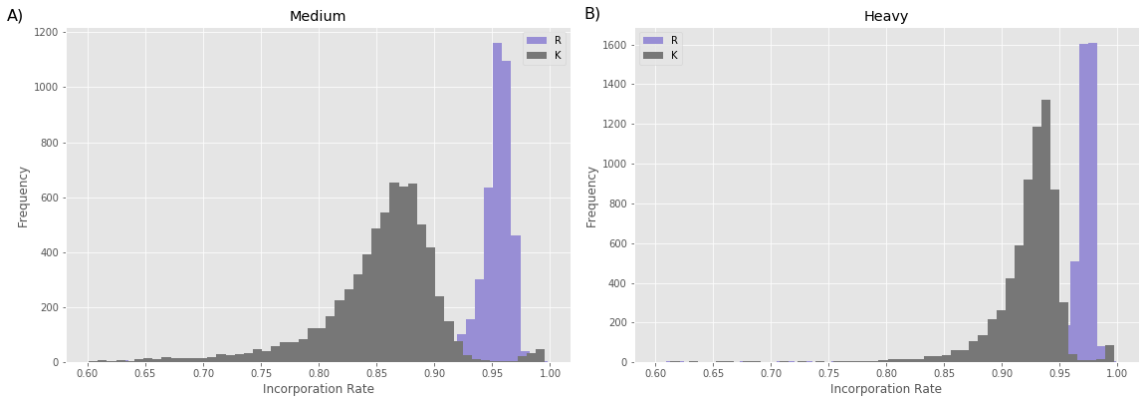
Completeness of isotope incorporation. The histograms show the proportions of isotope-labelled Lys (K) and Arg (R) residues (x-axis) against the frequency of tryptic peptides (y-axis) following the labelling to steady-state of bloodstream form cells with medium ( **A**) and heavy ( **B**) Lys and Arg.

Before the incorporation rate correction, the median of the normalized steady-state medium (R6K4) incorporation values prior the light (R0K0) chase were 0.88 for the BSF form and 0.95 for the PCF form. These apparently incomplete incorporation values ^29^ are a function of the isotopic purities of the labelled amino acids (99% for R10, R6 and K6 and 96% for K4) and, most likely, the acquisition of some light Arg and Lys from the fluid phase endocytosis and lysosomal degradation of serum albumen and other serum proteins from the culture media. Consistent with this view is the fact that isotopic Lys incorporation appears to be lower than that of Arg, reflecting the lower levels of Arg versus Lys in bovine serum albumen ( Figure 5). Further, as BSF parasites have a much higher endocytic rate than PCF parasites, it makes sense that the apparent under-incorporation is significantly greater in BSF parasites ^30^. To take into account these effects, we normalized the BSF values by a correction factor, as explained in the *Methods*.

**Figure 6.**
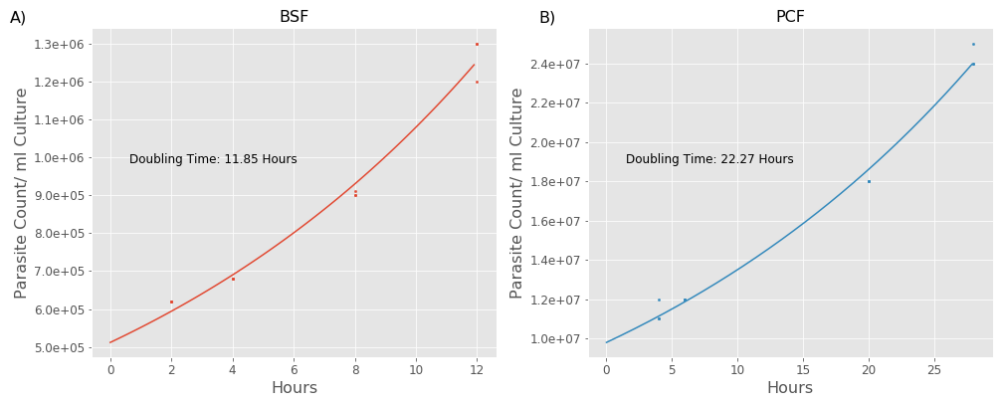
Parasite growth curves. Parasite counts per ml of culture (y-axis) are plotted against time for the triplicate bloodstream form (BSF) ( **A**) and procyclic form (PCF) (B) cultures used to determine protein turnover rates by label-chase. Growth Factors, used to correct for cell division during the course of the experiments, were obtained by dividing the parasite count values at each time point by the parasite count values at the point of resuspending the medium-labelled cells in light-chase medium (6.2×10 ^5^ cells/ml for BSF cultures and 1×10 ^7^ cells/ml for PCF cultures).

**Figure 7.**
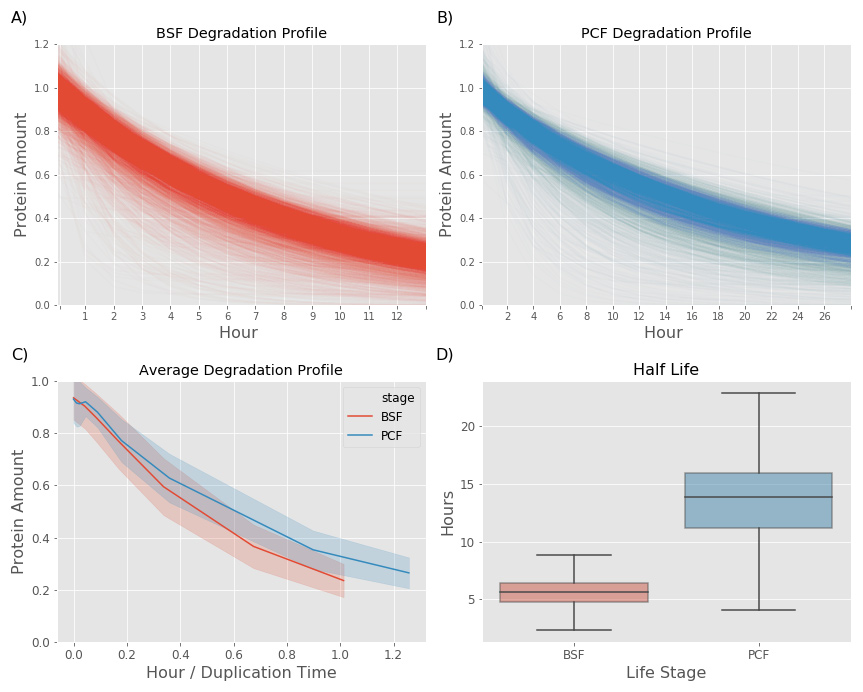
Protein turnover of bloodstream form (BSF) and polycyclic form (PCF) proteomes. ( **A**) The degradation profiles of BSF proteins. The y-axis reports the residual protein amount estimated from the quantitative proteomics data fitted to exponential decay models. ( **B**) The same as ( **A**) but for PCF proteins. ( **C**) The panel shows the average degradation profile of the BSF (red line) and PCF (blue line) proteins fitted to an exponential decay model. The shadow area surrounding the BSF and PCF lines represents the standard deviation. In this case, the x-axis reports the time points in hours of the time course divided by the measured doubling time of the BSF (11.8 hours) and PCF (22 hours) cells during the turnover experiments. ( **D**) The panel shows box plots the distribution of the protein half-lives for the BSF proteome (red) and the PCF proteome (blue).

**Figure 8.**
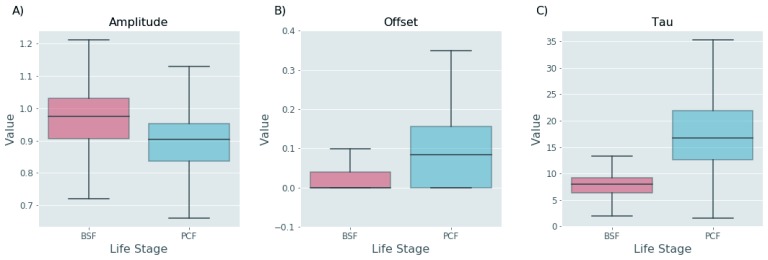
Exponential decay parameters. Box plots of the range of Amplitude ( **A**) Offset ( **B**) and Tau ( **C**) values determined from the fitting of data to exponential decay functions for bloodstream (red) and polycyclic form (blue) proteins.

**Figure 9.**
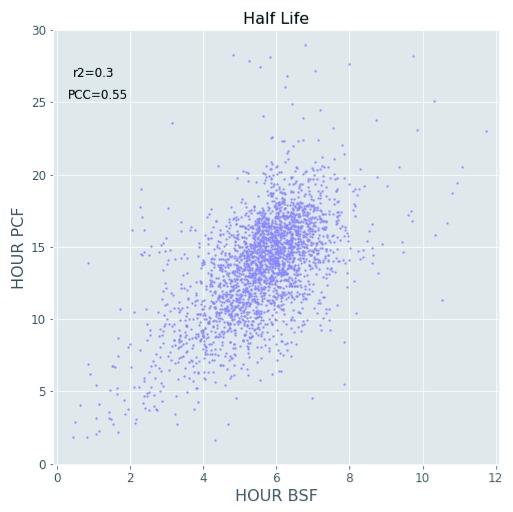
Correlation of protein half-lives between life-cycle stages. Scatter plot the protein half-life values computed for the bloodstream form (BSF) (x-axis) and the polycyclic form (PCF) (y-axis) life stages. The figure also shows the Pearson correlation coefficient (PCC) and the r ^2^ values.

Plotting the median protein degradation values against cell doubling time to normalise for the different doubling times of BSF and PCF cells ( Figure 6) suggests that protein turnover in BSF cells is significantly faster in this lifecycle stage ( Figure 7). This finding is supported by the distribution analysis of the fitted parameters. In the exponential decay model, the offset value (horizontal asymptote) can be used as a proxy for the residual amount of protein left after one round of cell division and the tau value can be used as a proxy for how fast the protein reaches this offset value. As both the offset and the tau values are, on average, smaller in the BSF relative to the PCF protein groups ( Figure 8), it is possible to conclude that the BSF proteome is more rapidly turned over than the PCF proteome, and this is also apparent from the computed median protein group half-lives corrected for the cell duplication ( Figure 7C). While higher rates of protein turnover might be expected in BSF cells from their 10°C higher growth temperature alone, it is also clear that other factors are also at play. Thus, the correlation of half-life values for proteins in common between BSF and PCF cells is quite low, with a Pearson correlation coefficient of 0.55 and r ^2^ value of 0.3 ( Figure 9). Such mechanisms might include factors leading to changes in the rate of protein synthesis, for example, mRNA stability and/or mRNA access to polysomes, and/or factors leading to changes in the rate of protein degradation, for example, ubiquitylation and proteasome-mediated proteolysis.

**Figure 10.**
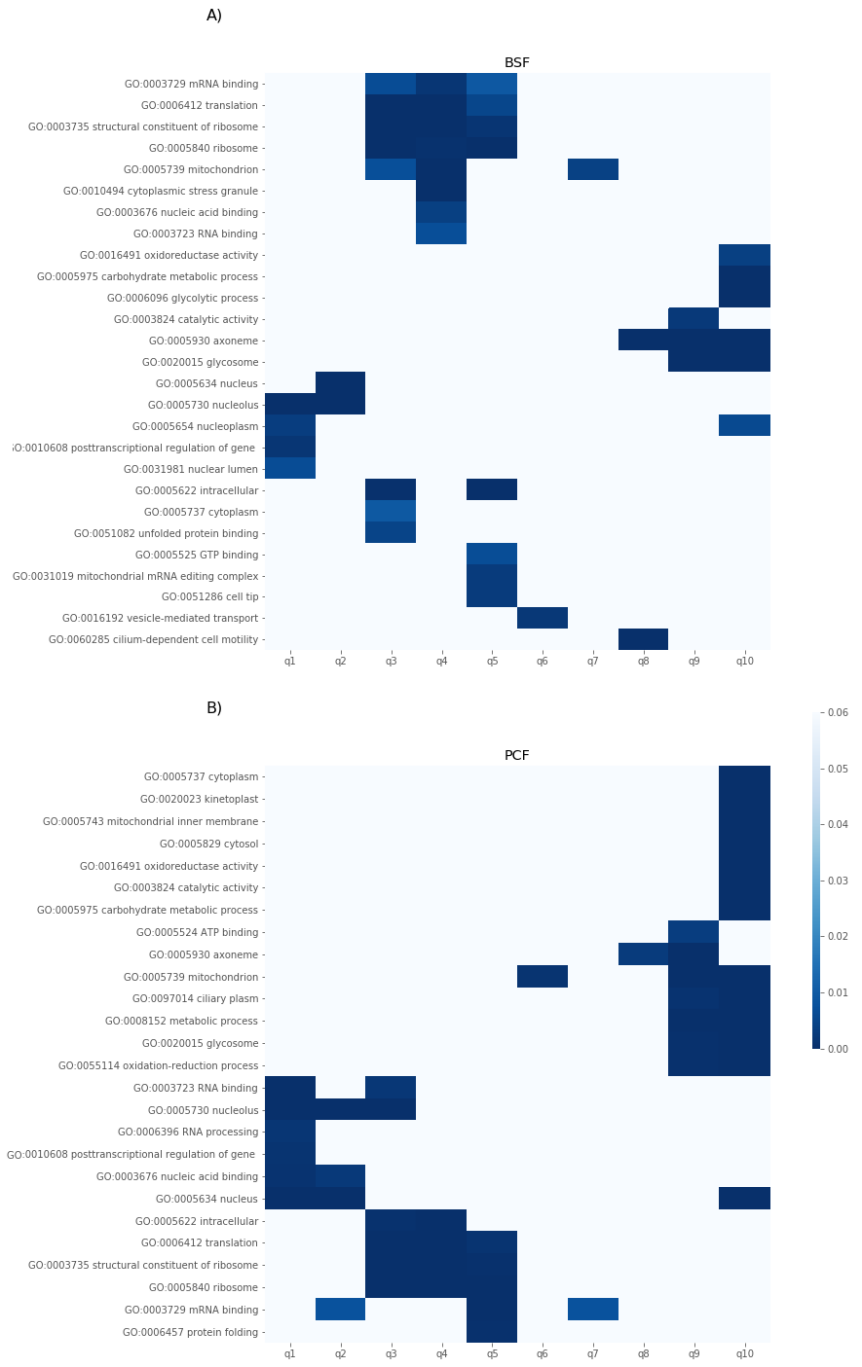
Protein half-life and Gene Ontology (GO) term enrichment analysis. The half-life values of the proteins fitted with an exponential decay model in bloodstream form (BSF) ( **A**) and polycyclic form (PCF) ( **B**) were divided into 10 quantiles (q1 to q10, x-axis). For each quantile, the enriched GO term (y-axis) and respective p-values are reported. The figure only includes the GO terms found enriched in fewer than four quantiles.

**Figure 11.**
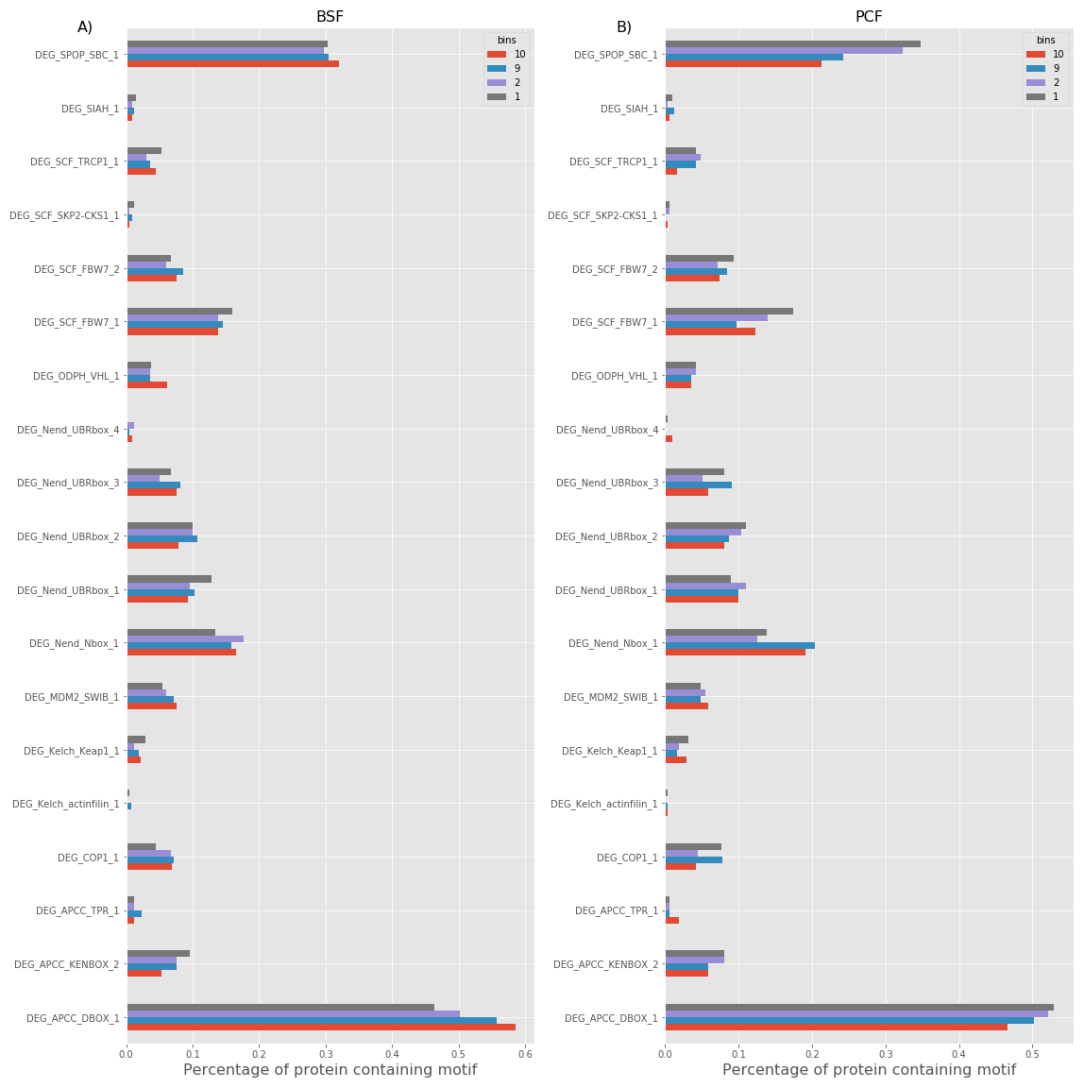
Linear motif. The percentage (x-axis) of proteins containing short degradation linear (or degron) motifs (y-axis) belonging to long half-life (1 ^st^ and 2 ^nd^) and short half-life (9 ^th^ and 10 ^th^) decile bins for the bloodstream form (BSF) ( **A**) and polycyclic form (PCF) ( **B**).

We next analysed potential links between cellular function and the stability of trypanosome proteins by binning the protein half-lives into decile groups and computing the GO term enrichment in each bin. A heatmap of the BSF and PCF GO annotations that were discovered in no more than 4 of the selected bins, with a p-value of 0.01 or less, is shown in ( Figure 10). In both lifecycle stages, the quantiles containing the shortest-lived proteins are enriched for terms that are related to regulation of gene expression and nucleolar localization, whereas the quantiles with the most stable proteins contain terms related to proteins and protein complexes localized to cellular compartments such as the mitochondria, the glycosome or the flagellum. The Trypanosome Go annotation does not contain data for short linear motifs. On the other hand, degrons (short linear motifs targeting proteins to degradation) have been found to influence the turnover in the human proteome ^31, 32^. For this reason, we wondered about the importance of degrons for the turnover of the *T. brucei* proteome. As illustrated in Figure 11, we could not find evidence for linear degradation motif enrichment in the primary sequences of the shortest-lived proteins (1 and 2 half-lives decile groups) relative to the more stable proteins (9 and 10 half-lives decile groups).

**Figure 12.**
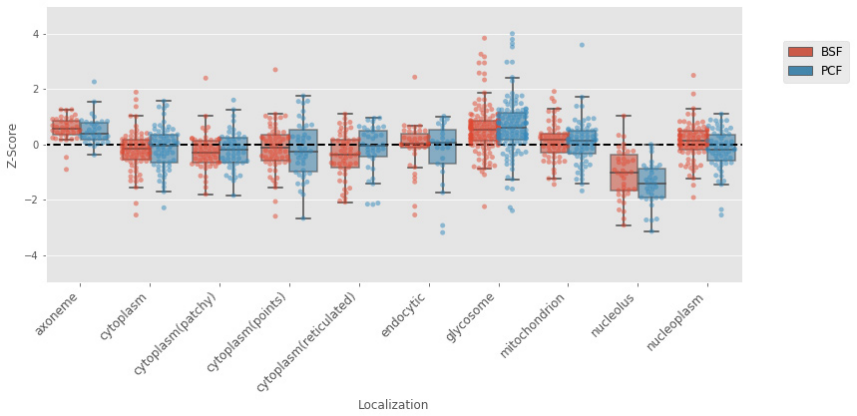
Protein half-life and protein localization analysis. The protein half-life values of the bloodstream form (BSF) (red) and polycyclic form (PCF) (blue) life stages were transformed to z-scores. The distributions of the z-score values (y-axis) are visualised with boxplots for each cell compartment (x-axis) derived from the annotations extracted from the TrypTag database.

To further analyse the relationship between protein localization and stability, we took advantage of the high-throughput imaging localization data of the TrypTag resource ^24^ deposited at TriTrypDB ^14^. From these data, we extracted the localization information for the 575 proteins annotated with single location descriptors from C-terminal tagging only (to minimise potential artefacts from disrupting N-terminal signal peptides). From this list, we removed cell compartments represented by <30 annotated proteins. Finally, we added the glycosomal proteome described in Guther *et al*. ^6^. We chose to add this experimentally determined glycosome subset of proteins (n=159) rather than rely on TrypTag localisation data for this organelle as many glycosomal proteins have C-terminal and/or N-terminal Peroxisomal Targeting Signal sequences ^11^. The assembled localisation dataset ( *Extended data*, Table 4 ^16^) was used to visualise the distribution of protein half-lives in each cell compartment. To better compare the BSF and PCF life stages, we transformed the protein half-lives into z scores. As illustrated in Figure 12, the glycosome and axoneme compartments contained proteins with generally higher stability than the average, while proteins targeted to the nucleolus were the least stable in both BSF and PCF.

**Figure 13.**
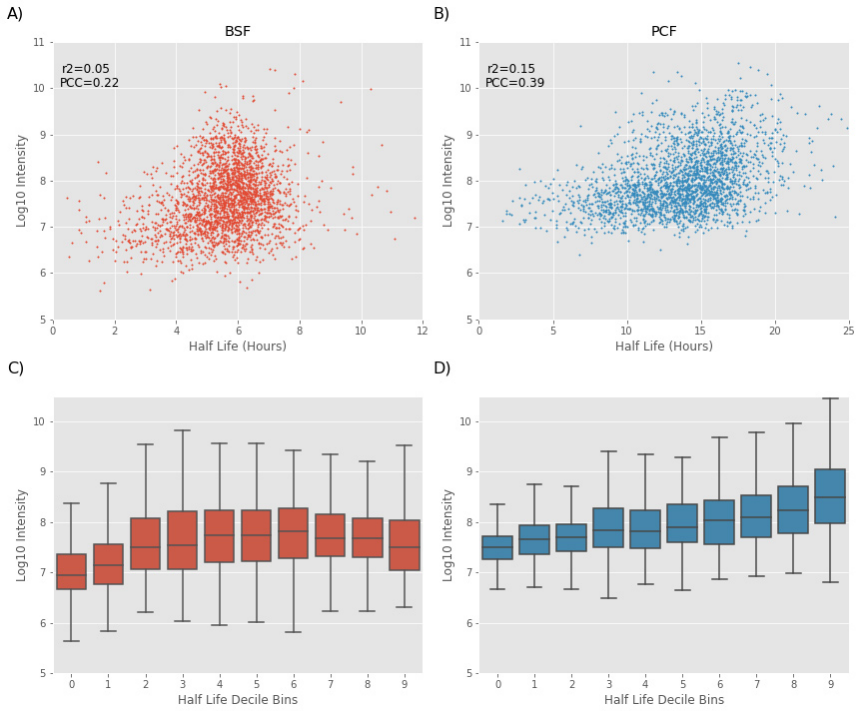
Protein half-life and protein abundance analysis. ( **A**) Scatter plot the protein half-life values (x-axis) and the protein abundance (y-axis) computed for bloodstream form (BSF) cells. The Pearson correlation coefficient (PCC) and the r ^2^ values are also shown. ( **B**) The same as ( **A**) but for polycyclic form (PCF_ cells. ( **C**) Box plots of protein abundance (y-axis) in each of the half-life decile bins (x-axis) for BSF cells. ( **D**)The same as ( **C**) but for PCF cells

The balance between protein synthesis and degradation defines proteostasis and protein abundance. This prompted us to asses any potential relationship between protein abundance and half-life. The scatter plots reported in Figure 13A and B shows that there is a poor correlation between abundance and protein half-life in both the BSF and PCF life stages. However, we noted that most of the proteins with longer half-lives have an intensity value greater than 1e8 in both BSF and PCF ( Figure 13A and B). Further, we observed that the protein abundance tends to increase with the protein half-life, with a more pronounced trend in the PCF relative to the BSF life stage ( Figure 13C and D).

### Turnover analysis of cell cycle regulated proteins

**Figure 14.**
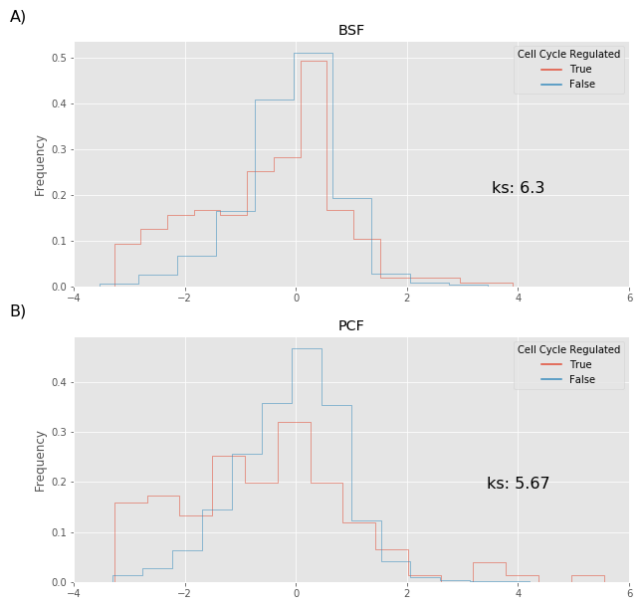
Protein half-life and cell cycle regulated proteins. The protein half-life values of the bloodstream form (BSF) ( **A**) and polycyclic form (PCF) ( **B**) life stages (x-axis) were transformed to z-scores. The distributions of the z-score values (y-axis) are shown in histograms for the total proteome (blue lines) and for the cell cycle regulated proteins (red lines). The figure reports the –log10 p value of the Kolmogorov-Smirnov statistic on 2-sample test (ks) for the z-score values of total proteome and the cell cycle regulated proteins.

We next sought to analyse the stability of proteins involved in the regulation of the cell cycle. To this end, we retrieved the cell cycle regulated proteins in PCF trypanosomes identified in a recent publication from our laboratory ^25^. As for the localization analysis, we decided to use the z-score to transform the protein residual amount and we could extract data for 197 and 120 proteins in the BSF and PCF respectively. As illustrated in Figure 14, the cell cycle regulated proteins are enriched for low z-score values in the PCF and BSF, suggesting that cell cycle regulated proteins are likely to have a faster turnover rate than the total proteome ( *Extended data*, Table 5 ^16^).

### Turnover analysis of protein complexes

**Figure 15.**
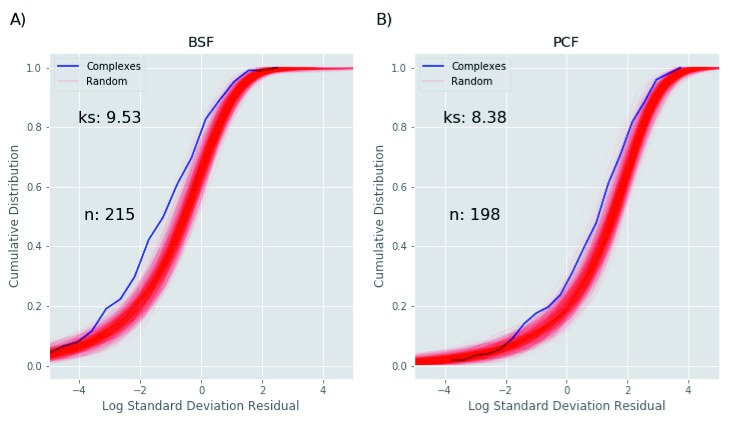
Protein half-life and protein complex analysis. The Figure compares the cumulative distribution (y axis) for the log10 transformed half-life standard deviation (x-axis) of bloodstream form (BSF) ( **A**) and polycyclic form (PCF) ( **B**) protein complexes (blue line) identified in 26, to the cumulative distribution of proteins randomly grouped into decoy pseudo-complexes, identically-sized to the protein complexes identified in 24 (red lines). The random complexes are assembled 100 times, each time with a different random seed. The –log10 p-value of the Kolmogorov-Smirnov statistic on 2-sample test (ks) and the number of protein complexes used for the analysis (n) are also shown.

To investigate the relationship between protein stability and membership of a protein complex, we took advantage of a recent publication from our laboratory aimed at the identification of soluble cytoplasmic protein complexes in *T. brucei*. In Croizer *et al*. ^26^, we defined a set of 234 high confidence protein complexes (Supplementary Table 2 of 13) based on protein elution profiles using two size exclusion and one ion exchange chromatography system. The residual protein value variance of the protein complex subunits shows a statistically significant difference, with the protein complexes having a smaller variance than seen for random protein complexes, for both BSF ( Figure 15A) and PCF ( Figure 15B). These findings support the hypothesis that proteins associating in the same complex are turned over at similar rates ^33^.

### Web resource

**Figure 16.**
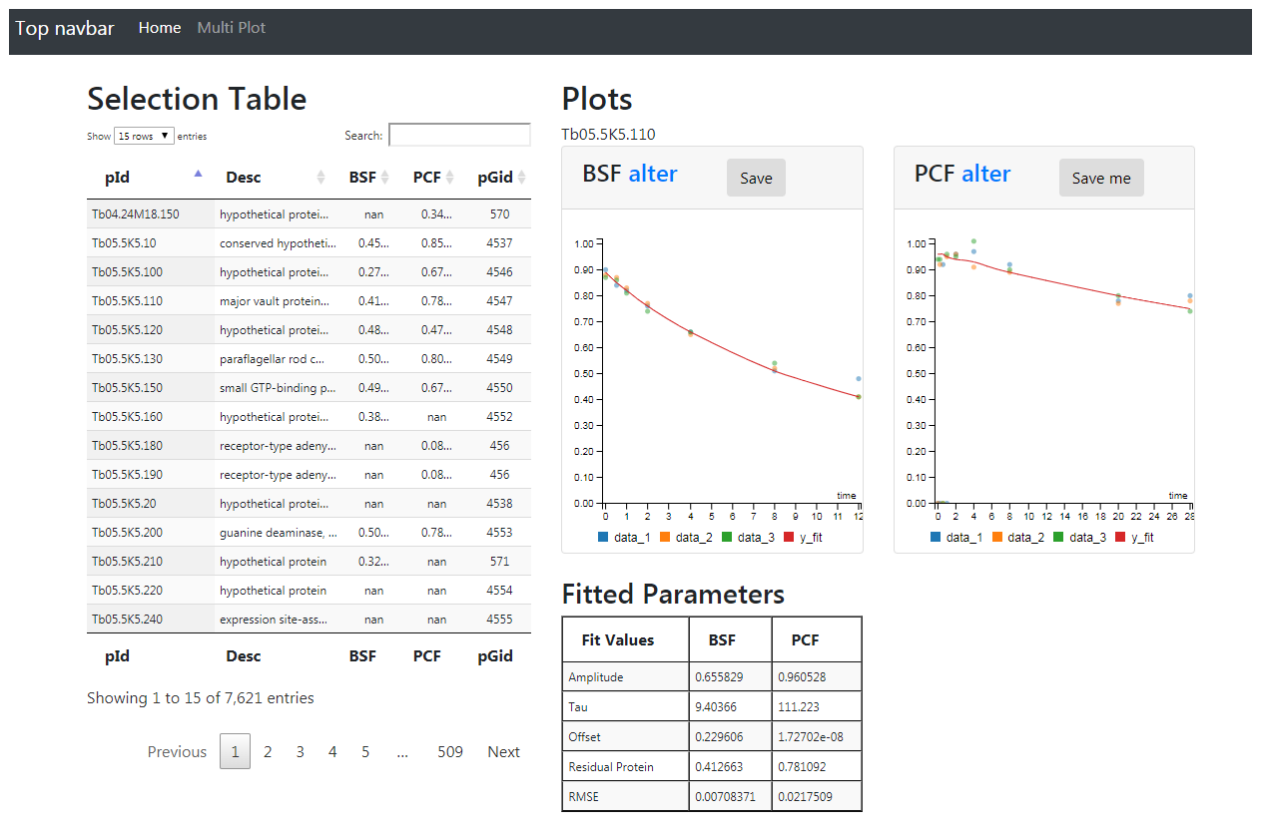
Web application. The figure displays the home page of the web application that allows users to compare the degradation profile of the bloodstream form (BSF) and polycyclic form (PCF) proteins identified in this work. The web application displays on the left side a Selection Table that lists the protein identifiers (pId), the protein descriptors (Desc), the half-life values for the BSF and PCF cells, and the MaxQuant protein group id (pGid). The table is searchable and sortable by all of the columns using the input box Search. A mouse click on a protein id loads new data and updates the plots and table on the right side of the web application. The upper right part of the web application displays two plots (Plots) reporting the normalized protein degradation values and fitted curves for the selected protein in BSF and PCF cells. The bottom right part of the web application reports the BSF and PCF parameters of the best fit (Amplitude, Tau and Offset), the protein half-life value (Half-Life) and the model error (RMSE) for the selected protein.

**Figure 17.**
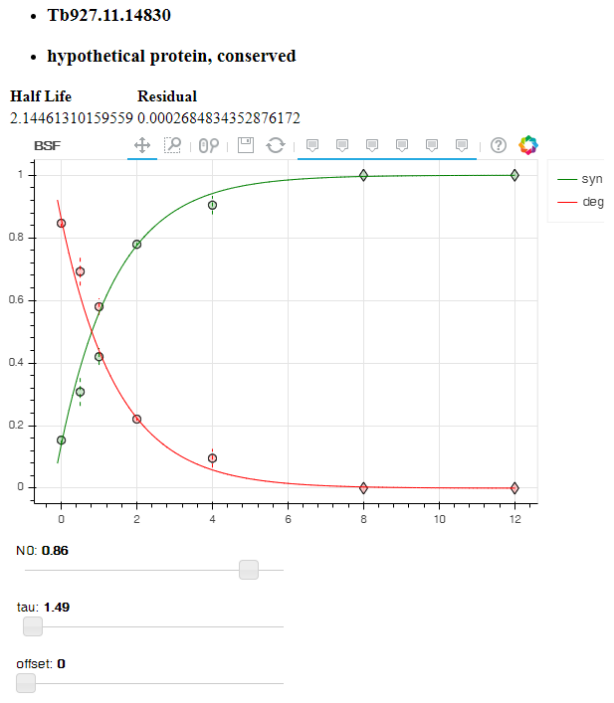
Web application: manual fit. The application plots degradation (red) and complementary synthesis (green) data. Each point represents the average of up to three experiments, and the vertical dashed lines represent the standard deviation. The data point markers are of three types: Round if there are data from 3 replicates, square if there are data from 2 replicates and diamond if there is only one replicate representing that time point. The user may adjust the parameters and observe the effects on curve fitting.

**Figure 18.**
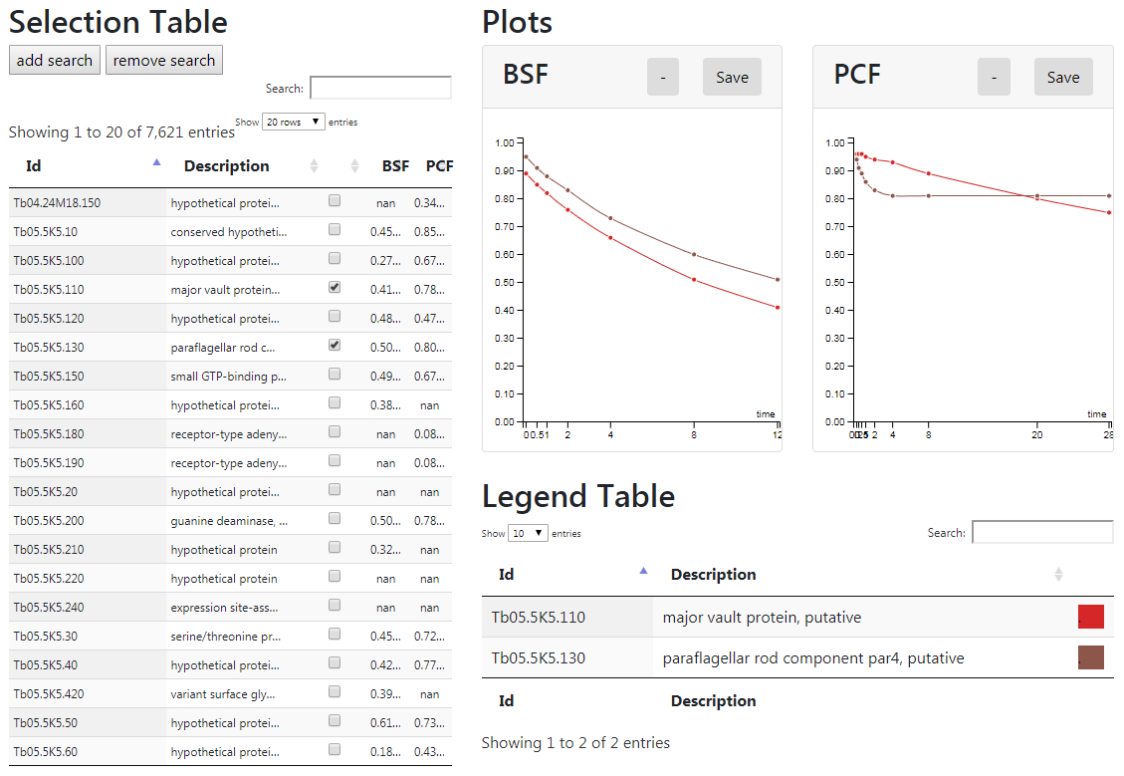
Web application: multiple comparison. The screen-shot shows how the turnover data of two (or more) proteins can be selected and displayed for both bloodstream form (BSF) and polycyclic form (PCF) cells.

All of the processed MS data and turnover analyses are freely available via a searchable web application that can be browsed at http://134.36.66.166:8082/turnover; code for the web application is available from GitHub and Zenodo ^27^. The web application displays two interactive search interfaces that visualise the turnover data for PCF and BSF cells, allowing a direct comparison between the two life stages. The first application at the home page allows the comparison of one protein at a time ( Figure 16). By clicking on any row of the Selection Table, the BSF and PCF normalised M/H degradation data points will appear in Plot panels on the right, with the best exponential fit for the data. The Selection Table is fully searchable by protein identifier and protein descriptions in the search field on the top of the table. A summary table (Fitted Parameters) at the bottom of the plots highlights the fitted parameters of the exponential decay model for both BSF and PCF. The link “alter” on the top of the BSF and PCF data plots opens a new window that contains interactive plots ( Figure 17). This new visualisation allows the modification of the exponential decay model parameters (amplitude, tau and offset) and updates the results of the newly fitted model. The second web application is found through the “Multi Plot” link and allows the comparison of multiple proteins at the same time ( Figure 18). The data to visualise can be uploaded with the Selection Table on the left of the application by ticking the boxes on the right of protein identifiers and descriptions. The Selection Table is fully searchable by protein identifier and protein descriptions with the search field on the top of the table. The protein(s) can be uploaded and visualised one by one or as a group. To search and upload a group of proteins, it is necessary to insert a search term in the search box and add the relevant proteins one by one.

## Discussion

This paper describes the first proteome-wide analyses of protein turnover in the BSF and PCF life-cycle stages of *T. brucei*. We hope this open data resource will be useful to the trypanosome research community.

The doubling times of the BSF and PCF cells in these experiments were approximately 12 h and 22 h, respectively ( Figure 6 and *Extended data*, Table 1 ^16^). The BSF doubling time is just beyond the upper end of the normal range for cultured parasites in our laboratory (8 to 10 h), whereas the PCF doubling time is longer than the normal range (12 to 14 h). We think that these relatively long doubling times are partly due to the necessary use of dialysed foetal bovine serum to perform the isotopic Arg/Lys labelling ^4^ and partly due to the effects of the unavoidable centrifugation step required to transfer the cells from medium- to light-isotope media to perform the chase, the stress of which seems to cause to growth arrest for about 2 h. Interestingly, the four PCF time points under 2 hours (0, 0.25, 0.5 and 1 h) show the lowest correlation compared all the other time points ( Figure 2 and Figure 3). Despite this lag-phase, the subsequent logarithmic growth suggests that the parasites are reasonably healthy for the majority of the chase period.

Despite those caveats, we think the data reported here are valuable. Some of the proteome-wide half-life values from our study appear to be in line with several specific examples reported in literature, whereas others differ. For example: BSF ISG75 and ISG65 are reported to have half-lives <3 h ^34, 35^, and our estimates for ISG75 (Tb927.5.360) and ISG65 (Tb927.2.3270) are 2.4 and 2.5 h, respectively. PCF CYC3ty (Tb927.6.1460) and CYC2ty (Tb927.11.14080) are reported to have half-lives >6 and >9 h, respectively ^36^, and our estimates (albeit in BSF) are 4.9 and 5.6 h, respectively. The turnover of the glycosomal protein fructose bisphosphate aldolase (Tb927.10.5620) has been determined with a pulse-chase experiment to be greater than 3 h ^37^ and our dataset shows a turnover rate of 7.8 h. A similar pulse-chase experiment has determined a rapid turnover (as short as 30 min) for aldolase and two other glycosomal proteins (D-glyceraldehyde-3-phosphate dehydrogenase and NAD-dependent glycerol-3-phosphate dehydrogenase) in PCF cells ^38^. Our data suggest a much longer half-life for those proteins (>7 h), as also suggested in a different study by Clayton in 1988 ^39^. The half-life of the main VSG (Tb427.BES40.22) has been reported in the order of 72 h ^40, 41^. However, our estimate of the main VSG half-life is 5.3 h, which is in good agreement with a recent determination of the VSG coat replacement (4.6 h) determined by flow cytometry ^42^.

The median half-lives of the BSF and PCF proteomes were 5.6 and 13 h, respectively, versus doubling times of 12 and 22 h, respectively ( Figure 6 and Figure 7). By contrast, recent studies on protein turnover in human and yeast cells showed median turnovers similar to the their respective cell doubling times ^17, 43, 44^. This suggest that in trypanosome protein replacement is not only driven by dilution due to cell division but also other by other active mechanisms, presumably including proteasome-mediated destruction. Interestingly, and consistent with this, proteasome inhibition has been shown to be highly toxic to trypansomatids ^45, 46^.

To get a better insight into the turnover of BSF and PCF proteome we divided the proteins into classes based on their relative turnover rates and we analysed those classes with a GO term enrichment strategy. A class of rapid turnover proteins is shared between BSF and PCF and is enriched for the “posttranscriptional regulation of gene expression” term ( Figure 10). The annotation of this set of proteins derives mostly from two high throughput screenings aimed at the characterization of the mRNA-binding proteome ^47, 48^. For this reason, it is not surprising that this class contains proteins with RNA binding domains. This finding suggests that the RNA binding proteins, which appear to be the primary modulators of gene expression in trypanosomes ^49, 50^, need to be switched on and off promptly as the case for the cell cycle-regulated proteins (see below). Also, the cell compartment “nucleolus”, contains rapid turnover proteins, as identified by both the GO enrichment and the TrypTag localization analyses ( Figure 10 and Figure 12). Several proteins targeted to this cell compartment are involved in ribosome subunit biogenesis ( *Extended data*, Table 5 ^16^) and previous studies have observed a higher turnover rate for nucleolar proteins in human and yeast cells as well ^17, 43^.

Another class of rapid turnover proteins in both BSF and PCF are cell cycle regulated proteins ( Figure 14). It is interesting to note that three of the identified BSF proteins (Tb927.11.15800: Tip Of Extending FAZ protein 1 or Cytokinesis initiation factor 1, Tb927.11.8220: aurora B kinase and Tb927.9.14290: Cytokinesis initiation factor 2) have been identified in a signal cascade that initiates cytokinesis. In particular, the phosphorylation of cytokinesis initiation factor 1 by polo-like kinase (Tb927.7.6310) targets it to the anterior tip of the new flagellum attachment zone filament, where it subsequently recruits Aurora B kinase to initiate cytokinesis ^51^. Localisation studies of these three proteins suggested a temporal relationship of appearance and co-localisation during the cell cycle, and our study provides evidence that this is achieved by a mechanism of synthesis and degradation. In a follow-up study, cytokinesis initiation factor 2 was found to interact with the cytokinesis initiation factor 1 ^52^. This study provided evidence that both overexpression or depletion of the cytokinesis initiation factor 2 inhibited cytokinesis, further suggesting that tight co-regulation of protein synthesis and degradation occurs for the proteins involved in this pathway.

From the GO term analyses, it is possible to extract a group of slow turnover proteins enriched for terms related to the flagella localization (axoneme, intraciliary transport particle, cilium motility, ciliary plasm) among which appear the components of the Rab family and intraflagellar transport (IFT) complex ( Figure 10 and *Extended data*, Table 2 ^16^). The Rab family contains membrane-associated proteins responsible for vesicle trafficking ^53^ while the IFT protein complex is responsible for the formation of the flagella and transport of protein from the base of flagella to their tip and back ^54^. The IFT protein complex itself contains members of the Rab family ^55^. The higher stability of these two protein classes compared to the proteins involved in cell cycle regulation might be explained by their localization. The membrane anchored proteins are generally recycled by an endocytosis mechanism ^56^. This mechanism might be slower than the ubiquitin-mediated proteasome degradation that acts on cytoplasmic proteins. On the other hand, the proteins of the IFT complex might be stabilized by their localization in the flagella, a very stable structure in *T. brucei* also during mitosis ^57^. This is supported by the observation that components of the axoneme are among the most stable proteins in BSF and PCF ( Figure 12).

The general hypothesis of localisation-dependent protein stability is further supported by the observed relatively slow turnover of proteins localized to the mitochondria and glycosomes ( Figure 10 and Figure 12). *T. brucei* contains a single mitochondrion that is replicated during cell cycle ^58^. It is likely that the proteins localized in this organelle are recycled/degraded by a different mechanism, i.e., mitophagy, with slower kinetics than experienced by cytosolic proteins ^59^. Many mitochondrial proteins have signal sequences at their N termini that are necessary and sufficient for import of the proteins into this organelle. The different turnover rates of individual mitochondrial proteins might then reflect the different rates at which these proteins are translocated to the organelle and their propensity for proteasome-dependent turnover prior to import. A similar situation could explain the range turnover rates seen for glycosomal proteins ( Figure 12): Thus, their relatively slow median turnover values would be consistent with peroxisomal autophagy (or ‘pexophagy’) as the principal mechanism of turnover for these organelles ^60– 62^, superimposed with protein-specific pre-import quality-control turnover mechanisms ^62^.

Previous turnover analyses in human cells showed that rapid turnover proteins contain specific sequence elements that may serve as degradation signals, such as PEST domains or cleavage sites and are enriched for disordered regions ^17, 31, 32^. More recently, it has been found that PEST domains or cleavage site degradation signals have little importance for the turnover of yeast proteins ^43^. Similarly, we did not find any evidence of enrichment for degrons in the BSF and PCF rapid turnover proteins ( Figure 11). It is possible to speculate that those degradation signals are less critical for protein turnover in unicellular eukaryotes such as yeast and trypanosomes, relative to multicellular eukaryotes.

Our analyses suggest that protein localisation and function contribute to determining the stability of the trypanosome proteome. On the other hand, the correlation between abundance and protein half-lives, as determined for the PCF and BSF life stages, shows that highly abundant proteins topically have long half-lives. This trend could be consistent with a model whereby steady-state protein levels in trypanosomes are controlled primarily by post-translational rates of degradation, rather than rates of synthesis.

Finally, we hope that the open access resource for accessing and querying the BSF and PCF protein turnover data at the URL http://134.36.66.166:8082/turnover ^27^ will prove useful to trypanosome researchers.

## Data availability

### Underlying data

EMBL-EBI PRIDE: Proteome Turnover in Bloodstream and Procyclic form Trypanosoma brucei Measured by Quantitative Proteomics. Accession number PXD007115; https://identifiers.org/pride.project:PXD007115.

Open access for interactive exploration of all proteomic data is provided via a web application: http://134.36.66.166:8082/turnover.

### Extended data

Zenodo: mtinti/wor_turnover: Turnover 1.0. https://doi.org/10.5281/zenodo.3417325 ^16^.

This project contains the following extended data:

- **Table 1. Parasite counts.** This table shows the parasite cell counts for each experimental replicate (Counts Replicate A, Counts Replicate B, Counts Replicate C) for the bloodstream form (BSF spreadsheet) and of the procyclic form (PCF spreadsheet) experiments. The table also reports the Growth Factors for each experimental replicate (Growth Factors A, Growth Factors B, Growth Factors C) obtained by dividing the parasite count for each chase time point by the parasite count at chase time t = 0.
- **Table 2. Bloodstream form (BSF) turnover analyses.** This table shows the data and data analyses for the BSF proteome. The column protein_id shows the top protein identification in the MaxQuant output. The columns amplitude, tau, offset report the parameter values end the columns amplitude_err, tau_err, offset _err report the error values from the fitting of the degradation data for that protein to an exponential decay function. The column half-life reports the protein half-life as computed from the fitted parameters. The column bins reports the protein half-life decile group to which the protein belongs. The colum exp_rmse reports the square root of the mean squared difference between the experimental values and the predicted values of the exponential model (RMSE). The column protein_groups reports the protein group from the MaxQuant output to which the top protein identification (protein_id) belongs. The column desc reports the protein descriptors of the protein groups from the MaxQuant output. The column used_for_analysis is a binary tag. It reports 1 if the protein passed the quality threshold (RMSE < 0.1) and 0 if it failed. The column GO reports the GO term annotations of the proteins in column protein_id. The rest of the columns report the normalized degradation SILAC values (M/H) of the 3 replicate experiments. The format of the those columns is (time point)_(replica id).
- **Table 3. Polycyclic form (PCF) turnover analyses.** This table shows the data and data analyses for the PCF proteome. The column protein_id shows the top protein identification in the MaxQuant output. The columns amplitude, tau, offset report the parameter values end the columns amplitude_err, tau_err, offset _err report the error values from the fitting of the degradation data for that protein to an exponential decay function. The column half-life reports the protein half-life as computed from the fitted parameters. The column bins reports the protein half-life decile group to which the protein belongs. The column exp_rmse reports the square root of the mean squared difference between the experimental values and the predicted values of the exponential model (RMSE). The column protein_groups reports the protein group from the MaxQuant output to which the top protein identification (protein_id) belongs. The column desc reports the protein descriptors of the protein groups from the MaxQuant output. The column used_for_analysis is a binary tag. It reports 1 if the protein passed the quality threshold (RMSE < 0.1) and 0 if it failed. The column GO reports the GO term annotations of the proteins in column protein_id. The rest of the columns report the normalized degradation SILAC values (M/H) of the 3 replicate experiments. The format of the those columns is (time point)_(replica id).
- **Table 4. Protein half-life and sub-cellular localization analysis.** This table reports the localization analysis for the protein identified in the TrypTag database. The column protein_id shows the top protein identification in the MaxQuant output. The column locs shows the name of the cell compartment locations taken from the TrypTag database. The columns BSFPCF report the protein half-life values as computed from the fitted parameters. The columns BSF Z score and PCF Z score report the z-scores for columns C and D, respectively. The column desc shows the protein descriptors of the protein groups from the MaxQuant output.
- **Table 5. Protein half-life of cell cycle regulated proteins analysis.** This table reports on the proteins identified as cell cycle regulated in 25. The column protein_id shows the top protein identification in the MaxQuant output. The column desc shows the protein descriptors of the protein groups from the MaxQuant output. The column Life Stage shows the trypanosome life stage (BSF or PCF). The column Z score shows the z-score for the half-life values.

Extended data, Tables 1–5 are available under the terms of the Creative Commons Attribution 4.0 International license (CC-BY 4.0).

## Software availability

**Analysis pipeline links to the raw data and code used to generate the paper figures are available at:** https://github.com/mtinti/wor_turnover.

**Archived code at time of publication:** https://doi.org/10.5281/zenodo.3417326 ^16^.

**The code used to run the web server is available at:** https://github.com/mtinti/wor_turnover_web.

**Archived code at time of publication:** https://doi.org/10.5281/zenodo.3428333 ^27^.

**Licence:** MIT.
