# Supplementary material for: Proteome turnover in the bloodstream and procyclic forms of Trypanosoma brucei measured by quantitative proteomics
Source: Wellcome Open Res. 2019 Oct 9;4:152. [Version 1] doi: 10.12688/wellcomeopenres.15421.1 (PMC6816455; doi:10.12688/wellcomeopenres.15421.1)
Supplement: Peer review history

# Peer review: Reviewer response for version 1

**DOI:** 10.21956/wellcomeopenres.16864.r36717

**Published:** 2019-10-28

**Authors:** Julius Lukes

**Affiliations:** 1 Institute of Parasitology, Biology Centre, Czech Academy of Sciences and Faculty of Science, České Budějovice, Czech Republic

This study comes from an excellent lab with long history of proteomic studies of trypanosomes. In this study they tried to establish the turnover of individual proteins in both life stages of *T. brucei*. Few if anyone else is better positioned to perform such a study. Along with the current TrypTag project, which established intracellular localization of all *T. brucei* proteins, this study positions African trypanosomes among the best established model eukaryotes, and it may qualify as perhaps the best studied protist.

 There are obvious limitations of the present study, such as the extremely long generation time of the procyclic stage (most likely due to technical issues), the fact that after filtering, the turnover could have been established for only about half of the predicted proteins (4194 + 3092 – 2600 = ~4700, if my estimation is correct) etc. However, such problems are inevitable and the wealth of data provided for the ~4700 proteins more than enough justifies indexing. Moreover, there is a good correlation with the limited data on protein stability available so far, and the results will be very important for studies of the VSG proteins, the same applies for the rapid turnover of RNA binding proteins in the light of the lack of a standard transcriptional control in these protists etc.

 The authors don’t address some surprising findings, such as the lack of a difference between the mitochondrion of procyclic and bloodstream trypanosomes, or substantial differences in this respect between their nucleoli, so if I may, I would encourage them to at least a bit extend the discussion by commenting on these novel and potentially important findings.    

 Minor issues to fix:

 Important typo – in the legends of most figures, there is polycyclic instead of procyclic.

 Page 17 – “…also other by other active mechanisms…“

 Page 17 – trypansomatids etc..

 There may be more, so careful proofreading is advised.

I confirm that I have read this submission and believe that I have an appropriate level of expertise to confirm that it is of an acceptable scientific standard.

# Peer review: Reviewer response for version 1

**DOI:** 10.21956/wellcomeopenres.16864.r36844

**Published:** 2019-10-23

**Authors:** Christine Clayton

**Affiliations:** 1 Department of Microbiology, University of Heidelberg, Heidelberg, D-69120, Germany

When I was asked to review this article I had already read it and also cited it in a manuscript revision which will be submitted by the end of this week. I cannot judge the technical or statistical aspects but the provision of half-life information for all detected proteins is incredibly useful. The online application is really easy to use, replicates are visible, and Tables are provided for automated lookup as well. For future analyses, the code is provided - this could be invaluable to detect manipulations that selectively affect turnover of some proteins.

 Minor point - I was impressed that the authors found several previous individual turnover measurements since these are usually hidden as an afterthought within manuscripts talking about something else.

I confirm that I have read this submission and believe that I have an appropriate level of expertise to confirm that it is of an acceptable scientific standard.

# Peer review: Reviewer response for version 1

**DOI:** 10.21956/wellcomeopenres.16864.r36719

**Published:** 2019-10-15

**Authors:** Keith R. Matthews

**Affiliations:** 1 Institute for Immunology and Infection Research, School of Biological Sciences, University of Edinburgh, Edinburgh, UK

This is a technically accomplished study of protein turnover in African trypanosome parasites that will be of enormous long-term value to the trypanosome research community. The work uses stable isotope labelling to monitor the decay of proteins in two life cycle stages of the parasite in culture and then analyses the resulting datasets globally and with respect to some cellular organelles providing an overview of general and localised turnover. The work is of high quality, thorough and very nicely presented. Of course, more in depth analysis of specific protein groups or processes could be carried out but this is not necessary or appropriate at this stage - the follow-on work from other labs using this data will build this added-value in. I have only a few comments which are largely reflections of points already raised by the authors themselves, plus a tiny technical query.

1. The correlation between decay rates in bloodstream and procyclic forms is surprisingly low. Of course, there are stage-specific processes and regulation (as suggested by the authors) but regardless I would have expected better correlation of, at least, proteins involved in the most fundamental processes. Clearly, this can be explored further in future to explore the common and differentially regulated protein subsets to uncover some new biology, perhaps.
2. It might have been valuable to have a correlation between mRNA abundance (and decay) and protein abundance (and decay). I think there's plenty of evidence that these can correlate poorly but it would be valuable to understand how these regulatory levels interact. Since several mRNA datasets (including decay studies) exist, this could be a useful follow up study.
3. The data are all publicly accessible but most use will be derived through availability in TriTrypDb (or at least as a link from the gene code in TriTryp DB to the web application for viewing decay rates). I assume the authors are coordinating with TriTrypDB curators to incorporate this information into forthcoming TriTrypDB data releases?

A minor technical point relates to the cell lines used. The bloodstream form strain used is 427. I assume the procyclic forms are of the same strain? As it stands the PCF are described as 29.13.6 cells but I think these assignations relate to plasmid insertions not the parasite strain itself.

I confirm that I have read this submission and believe that I have an appropriate level of expertise to confirm that it is of an acceptable scientific standard.
